# Anti-xa guided enoxaparin thromboprophylaxis is associated with less thromboembolism than fixed dose dalteparin in trauma patients admitted to intensive care

**DOI:** 10.1007/s00068-025-02768-z

**Published:** 2025-02-07

**Authors:** Ahmad Kloub, AbuBaker Alaieb, Ahad Kanbar, Suha Abumusa, Fajer Alishaq, Yazan Hinawi, Naushad Ahmad Khan, Mohammad Asim, Tarik Abulkhair, Ayman El-Menyar, Hassan Al-Thani, Sandro Rizoli

**Affiliations:** 1 Department of Surgery, Trauma Surgery, Hamad Medical Corporation, Doha, Qatar; 2 Clinical Research, Trauma and Vascular Surgery, Hamad Medical Corporation, Doha, Qatar; 3 Weill Cornell Medical College, Doha, Qatar

**Keywords:** Anti-xa, LMWH, Venous thromboembolism, Thromboprophylaxis, Trauma

## Abstract

**Background:**

Venous Thromboembolism (VTE) is a common, preventable complication in trauma. Low-molecular-weight heparin (LMWH) is recommended for VTE prophylaxis (VTEp). We investigated whether switching from fixed-dose dalteparin to anti-Xa-guided enoxaparin prophylaxis reduces VTE without increasing the risk of bleeding among hospitalized trauma patients.

**Methods:**

This observational study compared injured patients admitted one year before (pre-P) and after (post-P) implementing a new VTEp protocol. The protocol was introduced as a performance improvement project (subcutaneous enoxaparin 30 mg twice daily), with dose calibration to peak plasma Anti-Xa level measured after the 3rd dose. The primary outcomes were the rate of VTE and bleeding.

**Results:**

After protocol implementation (post-P), 305 patients were compared to 350 pre-protocol patients (pre-P). Anti-Xa levels were measured in 83% of post-P and none in the pre-P. 40% had low levels of anti-Xa, suggesting inadequate prophylaxis, and enoxaparin doses were accordingly increased. 51% attained the desired anti-Xa levels, 9% had higher levels, and LMWH doses were subsequently reduced. VTE incidence after protocol implementation decreased from 4 to 1.3% (OR 0.31; 95% CI 0.1–0.9, *P* = 0.03) without increasing the bleeding rate. The time intervals between two consecutive PE events were significantly longer after protocol implementation. Among TBI patients, the rate of VTE was lower. However, it did not reach statistical significance. 75% of patients with VTE had low anti-Xa levels, while 20% of those with bleeding had high anti-Xa levels.

**Conclusion:**

Among adult patients in the trauma ICU, compared to a fixed dose dalteparin, enoxaparin prophylaxis with dose calibration according to peak anti-Xa levels was associated with lower VTE rates without increasing the risk of bleeding. About 40% of patients who received initial enoxaparin doses of 30 mg twice daily had anti-Xa levels suggestive of inadequate prophylaxis. Calibrating LMWH dosing may improve VTEp following traumatic injury.

## Introduction

Following the initial phase of trauma, a hypercoagulable state predisposes patients to venous thromboembolism (VTE), namely, pulmonary embolism (PE) and deep venous thrombosis (DVT) [1, 2]. Although the VTE rate varies among studies, PE continues to be a major cause of potentially preventable deaths in trauma patients after the first twenty-four hours post-injury [3]. 

VTE prophylaxis (VTEp) is currently recommended for trauma patients [4]. There has been a debate regarding the superiority of low molecular weight heparin (LMWH) over unfractionated heparin (UFH). A recent systematic review and meta-analysis have shown that LMWH is more protective against DVT in trauma than UFH and may decrease the incidence of PE and mortality. This paper did not reach a conclusion regarding adverse events [5]. 

LMWH inhibits coagulation factor Xa and has predictable pharmacokinetics and pharmacodynamics, which precludes the need for routine drug monitoring [6]. Still, monitoring by.

plasma Anti-Xa activity had previously been recommended for high-risk situations like pregnancy, extremes of body weight, and in patients with renal insufficiency [7]. Recently, using anti-Xa testing for VTEp monitoring in trauma has gained increasing interest.

In trauma, the most used LMWHs are enoxaparin and dalteparin. Enoxaparin is the main medication used when anti-Xa-guided therapy is utilized [8, 9]. In this study’s cohort, the rate of VTE was notably high at 4% before implementing the new VTEp protocol. Therefore, prophylaxis was switched from fixed-dose dalteparin dosing to anti-Xa-guided enoxaparin prophylaxis, aiming to decrease the incidence of VTE.

In this study, we hypothesized that, among trauma patients admitted to the TICU, transitioning the venous VTEp protocol from a fixed-dose dalteparin regimen to an anti-Xa-guided enoxaparin regimen will result in a reduction in the incidence of VTE without increasing the post-prophylaxis bleeding events.

## Patients and methods

All injured adult patients admitted to the trauma intensive care unit (TICU) at Hamad Trauma Center (HTC) were prospectively enrolled for a 12-month period from June 2022 to May 2023 (post-P). HTC is the only adult level 1 trauma center in the state of Qatar. Serving a population of approximately 2.7 million, the center operates with a policy of delivering care at no cost to patients, ensuring equitable access to advanced trauma services.

The new VTEp protocol was implemented as a performance improvement project on June 10, 2022. Enoxaparin dosages in the study group (post-P) were calibrated based on peak plasma anti-Xa levels measured after the administration of the third enoxaparin dose. The outcomes of this group were compared with a historical control of trauma patients (pre-P). The pre-P group was admitted to the TICU over a one-year period directly before the new protocol implementation (June 2021- May 2022). For the pre-P group, the VTEp protocol was fixed dose dalteparin given in a single daily dose. The total number of TICU admissions during these two years was 1064 patients.

The HTC Registry and electronic medical records were reviewed. Additionally, all performance improvement reports auditing VTE cases and suspected bleeding episodes were examined. The HTC registry is regularly validated at the internal and external levels. It reports quarterly to the Trauma Quality Improvement Program–American College of Surgeons (TQIP-ACS).

**Fig. 1:**
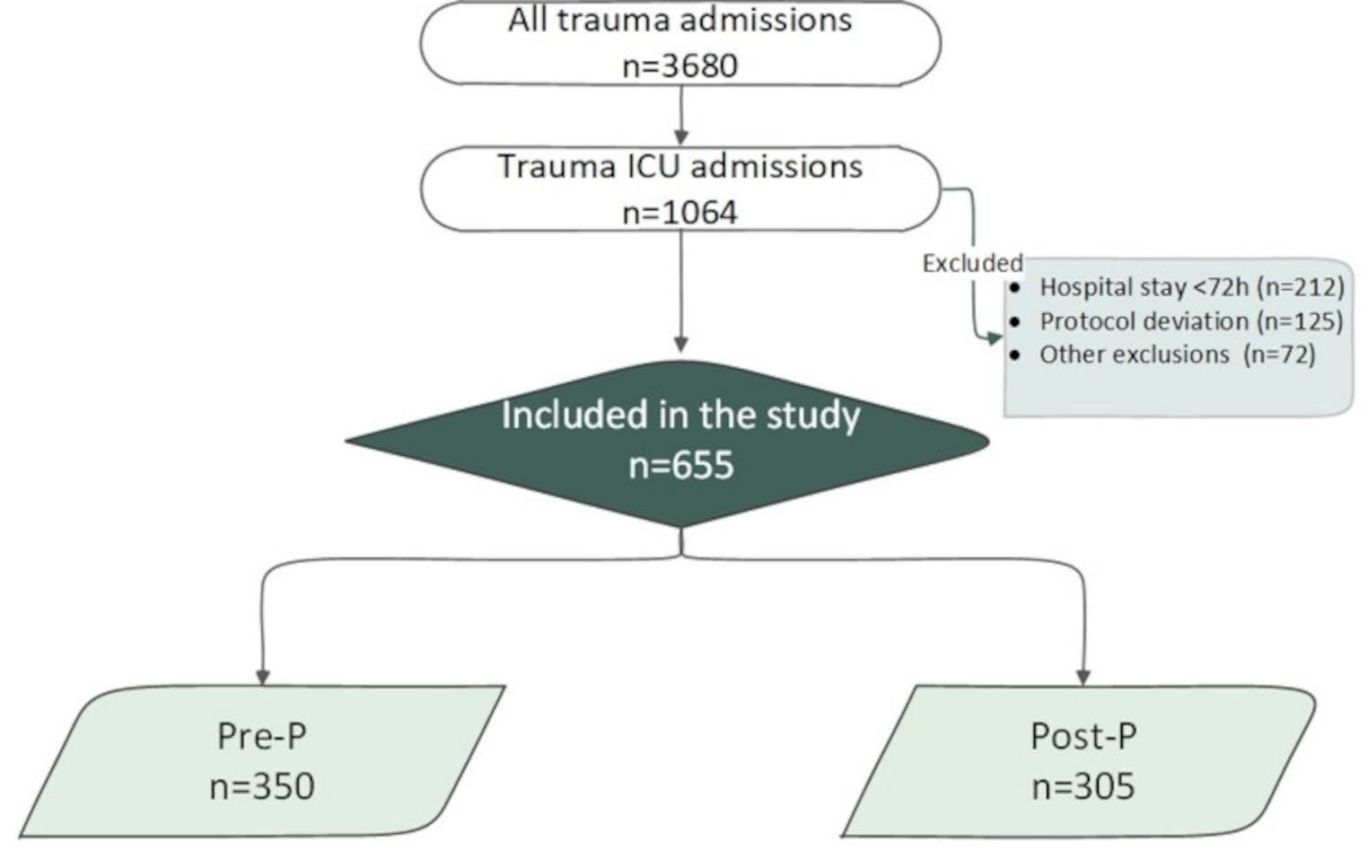
Flow chart for the study design

The exclusion criteria were patients who stayed < 72 h at the hospital, pediatric patients, pregnant, renal failure, a known history of malignancy, patients admitted while on anti-coagulation or anti-platelets therapy, presence of a pulmonary artery blood clot within 24 h of admission, and patients transferred from another facility. Figure 1 shows the study design.

All patients had lower limb pneumatic sequential compression devices unless the condition of the lower limbs precluded device application. As for chemoprophylaxis, the pre-P received once daily dalteparin dosing (5000IU and 7500IU for patients with a body mass index (BMI) of 40 or more), which was started at the discretion of the trauma attending. For all patients with TBI, the opinion of the neurosurgeons was considered. There was no anti-Xa monitoring in the pre-P group. The post-P were the patients who complied with the new protocol after its implementation.

### The new VTEp protocol

Between June 10, 2022, and May 10, 2023, adult patients admitted under the trauma intensive care service were prospectively enrolled into the post-P cohort upon the initiation of enoxaparin prophylaxis. All enrolled patients were administered an initial prophylactic dose of 30 mg enoxaparin subcutaneously twice daily. The dose was weight-adjusted to 0.5 mg/kg in patients with BMI ≥ 30, except in cases of TBI, age above 65, or creatine clearance of 30–60 ml/m. VTEp for non-TBI patients was initiated 24–48 h after admission. In TBI patients, VTEp was started 24–48 h post-admission only if the 24 h follow-up brain CT scan was stable, and no neurosurgical intervention was planned within 12 h. Otherwise, VTEp was held for another 24 h, followed by a repeat CT scan. The neurosurgical team always cleared the initialization of VTEp in TBI patients.

VTEp was withheld in cases of bleeding, suspected bleeding, and suspected or confirmed coagulopathy. Interruptions of VTEp for any invasive procedure or surgery were minimized.

The enoxaparin dose adjustment was based on the peak anti-Xa level measured 3–5 h after the third consecutive dose. The target anti-Xa range was 0.2 to 0.4 IU/ml.

**Fig. 2:**
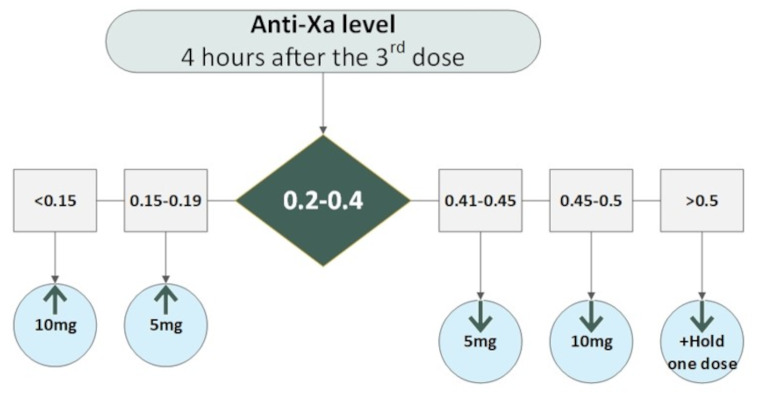
Enoxaparin dose titration per Anti-Xa level

Titration of dosage was based on a predetermined strategy. For peak anti-Xa levels < 0.15 IU/ml, the enoxaparin dose was increased by 10 mg/dose. For peak anti-Xa levels > 0.45 IU/ml, the enoxaparin dose decreased by 10 mg/dose. For anti-Xa levels 0.15–0.19 or 0.41-045, the dose was adjusted by 5 mg. For Anti-Xa level > 0.5, one dose of enoxaparin was held, then restarted with a smaller dose. Anti-Xa level was followed up after adjustments. Enoxaparin continued until the patient was discharged from the hospital (Fig. 2).

Variables collected included demographic characteristics, BMI, history of chronic illness, previous venous thromboembolism, mechanism of injury, prehospital time, injury severity score (ISS), head abbreviated injury score (AIS), spinal cord injury, unstable spinal fractures, pelvic and long bone fractures, and laboratory parameters on admission. Also recorded the administration of blood products, prothrombin complex concentrate, fibrinogen concentrate, Tranexamic acid, intubation, femoral line insertion., ventilatory days, length of stay in the ICU, time of initiation of enoxaparin, anti-Xa levels, number of dosing interruptions, total hospital stays, and mortality.

Outcome data were the incidence of VTE, PE, DVT, or concomitant PE and DVT, as well as significant bleeding episodes that occurred after initiating VTEp. Diagnosis of DVT was based on duplex ultrasonography, and diagnosis of PE on chest computed tomographic angiography (CTA) of the pulmonary artery upon clinical suspicion; sub-segmental PE was excluded. Proximal Lower limb DVT screening was performed by trauma intensivists using bedside compression ultrasound along with standard Duplex confirmation for any positive or suspicious screening result. Screening bedside compression ultrasound was performed routinely in all TICU patients on the day of admission, then on days 3,7,14, 21, and 28 post admission. Screening was stopped when patients became fully ambulatory, fully anti-coagulated for any reason, or were discharged from the hospital before day 28. Upper limb and distal lower limb DVTs (below the trifurcation of the Popliteal vein) were excluded. Significant bleeding was defined as any bleeding episode requiring blood transfusion, surgical or radiological intervention, Hemoglobin drop of more than 2 gram/dl, new intracranial bleeding or progression of a previous bleed, paraspinal or intraocular bleed. Compliance with timely sampling of the anti-Xa blood level was measured. Anti-Xa levels were studied in relation to the patient’s weight, BMI, creatinine clearance, and Injury severity score (ISS).

The institutional medical review board (Medical Research Center, MRC) (MRC-01-23-872) approved the study protocol at Hamad Medical Corporation, Qatar. A waiver of consent was granted as the data were collected retrospectively and anonymously without direct contact with patients.

#### Statistical analysis

Data were expressed as numbers, percentages, and means with standard deviation or medians with range for categorical variables, whenever appropriate. Demographic, clinical, and laboratory parameters and outcomes were compared among the pre-P and post-P groups. Subgroup analysis was also performed among severe TBI patients (AIS 3–5) in the pre-P and post-P groups. The chi-square test was performed to analyze differences in categorical variables between groups, and the Fisher exact test was used when observed cell values were less than 5. Continuous variables were compared using Student’s t-test for two groups for parametric data. The Mann–Whitney U-test was used for non-parametric data whenever applicable.

Furthermore, data were also expressed as odds ratio (OR) and 95% confidence interval (CI). A post hoc power analysis was conducted to determine the study’s statistical power. Two-tailed *p* < 0.05 was considered as statistically significant. Data analysis was conducted using the Statistical Package for Social Sciences version 21 (SPSS Inc., Chicago, IL).

The Shewhart T-chart was utilized to monitor the time intervals between consecutive PE events and to assess trends before and after implementing the new protocol. The T-chart, designed for rare events, plots the time intervals (Y-axis) against the dates of events (X-axis). Trend is statistically significant when more than six consecutive data points fall on the same side of the centerline, two out of three successive points on the same side of the centerline are farther than two σ, or a single point farther than the upper control limit [10].

## Results

The study included a total of 655 patients with traumatic injuries admitted to the TICU and were included in the final analysis. Initially the study excluded patients who stayed less than 72 h at the hospital (*n* = 212), age less than 16 (*n* = 8), pregnancy (*n* = 6), creatinine clearance less than 30 ml/m. (*n* = 12), a known history of cancer (*n* = 2), patients admitted while on anti-coagulation (*n* = 2) or anti-platelets therapy other than aspirin (*n* = 10), presence of a pulmonary artery blood clot within 24 h of admission (*n* = 3), extracorporeal membrane oxygenation (ECMO) for indications other than PE (*n* = 2), and patients transferred from another facility (*n* = 27). During the post-implementation phase, 29% (125 of 430) of TICU patients were excluded from the final analysis for not adhering to the new protocol and continuing the prior dalteparin-based regimen. After exclusions, the pre-P and post-P groups were 350 and 305, respectively (Fig. 1). The two cohorts were comparable in terms of age, sex, and BMI (Table 1). The mean age of the patients was 36 years, and 94% were male. The ISS was similar between the groups (19.9 ± 9.9 vs. 19.1 ± 10.2, *p* = 0.36). However, the head Abbreviated Injury Scale (AIS) score was significantly lower in the post-P group (3.6 ± 0.9 vs. 3.8 ± 0.9, *p* = 0.02), and had a higher rate of pelvic fractures (26.6% vs. 15.1%; *p* = 0.001), and spinal cord injuries (33.8% vs. 19.7%, *p* = 0.001).

**Table 1:** Comparative analysis for Pre-P and Post-P groups

Parameters	Pre-P (*n* = 350)	Post-P (*n* = 305)	*P*-Value
Age	36.2 ± 14.3	36.1 ± 13.3	0.87
Gender			
Male	330 (94.3%)	285 (94.7%)	0.82
Female	20 (5.7%)	16 (5.3%)	
Body Mass Index (BMI)	26.8 ± 5.3	26.4 ± 4.6	0.22
**Laboratory parameters at Baseline**			
Hemoglobin	13.0 ± 2.1	12.9 ± 1.9	0.54
Platelets	247 ± 75.3	246.8 ± 76.9	0.92
INR	1.09 ± 0.1	1.13 ± 0.2	0.001
PTT	25.9 ± 5.0	25.1 ± 5.2	0.04
Fibrinogen	2.7 ± 1.1	2.8 ± 1.9	0.43
Lactate	2.4 ± 1.8	2.5 ± 1.5	0.52
Base Excess (BE)	-2.2 ± 4.5	-2.8 ± 4.6	0.07
ROTEM baseline abnormal	82 (25.2%)	69 (23.5%)	0.62
TXA administered	77 (22.0%)	77 (25.6%)	0.28
PCC administered	5 (1.4%)	3 (1.0%)	0.61
Fibrinogen administered	45 (12.9%)	48 (15.9%)	0.26
Blood transfusion within 24 h			
pRBC	53 (15.1%)	41 (13.4%)	0.53
Fresh frozen plasma (FFP)	19 (5.4%)	16 (5.2%)	0.91
Platelet	16 (4.6%)	10 (3.3%)	0.39
Injury Severity Score (ISS)	19.9 ± 9.9	19.1 ± 10.2	0.36
Head AIS	3.8 ± 0.9	3.6 ± 0.9	0.02
Severe TBI	161 (46%)	127 (42%)	0.26
Spinal cord Injury	69 (19.7%)	103 (33.8%)	0.001
Solid organ injury	80 (22.9%)	68 (22.3%)	0.86
Unstable Spinal Fracture	33 (9.4%)	23 (7.5%)	0.38
Pelvic fracture	53 (15.1%)	81 (26.6%)	0.001
Long bone fracture	85 (24.3%)	77 (25.2%)	0.77
Prehospital time	88.8 ± 55.4	96.7 ± 88.2	0.17
SBP at admission			0.49 for all
< 90	32 (9.1%)	23 (7.6%)	
≥ 90	318 (90.9%)	278 (92.4%)	
Previous history of VTE	1 (0.3%)	0 (0.0%)	0.35
Pre-admission Aspirin	5 (1.4%)	7 (2.3%)	0.40
Diabetes Mellitus (DM)	42 (12.0%)	41 (13.4%)	0.57
Hypertension	28 (8.0%)	38 (12.5%)	0.05
Coronary Artery Disease	7 (2.0%)	14 (4.6%)	0.06
Positive pressure ventilation (*n* = 276)			
≤ 5 days	97 (61.8%)	80 (67.2%)	0.35 for all
> 5 days	60 (38.2%)	39 (32.8%)	
Ventilator days	3 (1–87)	3 (1–76)	0.76
Femoral line insertion	23 (31.1%)	10 (21.7%)	0.26
LOS in ICU	4 (3–75)	3 (3–46)	0.01
Total hospital LOS	12 (3–75)	13 (3-237)	0.82
In-hospital mortality	12 (3.4%)	6 (2.0%)	0.25
Time to initiate chemoprophylaxis (hours)	49.3 (43.9–54.6)	43.7 (40.9–46.4)	0.15
VTEp interruptions	2 (1–33)	2 (1–17)	0.93

INR: The international normalized ratio; PTT: partial thromboplastin time; TBI: Traumatic brain injury; AIS: Abbreviated Injury Scale; DVT: Deep vein thrombosis; LOS: Length of stay; ICU: Intensive Care Unit; ROTEM: Rotational Thromboelastometry; VTEp: Venous thromboembolism post-implementation protocol; pRBC: packed red blood cells

Additionally, the ICU length of stay (LOS) was significantly shorter in the post-P group (3 days [range 1–46] vs. 4 days [range 1–75], *p* = 0.01). However, total hospital LOS was similar between groups (13 days vs. 12 days, *p* = 0.82), as was in-hospital mortality, 2% in post-P and 3.4% in pre-P, *p* = 0.25.

Initial laboratory values were also similar except for post-P, which had a higher mean INR (1.13 vs. 1.09; *p* = 0.001) and lower mean PTT (25.1 vs. 25.9; *p* = 0.04). Other clinical outcomes were comparable, including ventilation duration, prehospital times, chemoprophylaxis timing, and blood transfusion requirements. A marginal increase in hypertension (12.5% vs. 8.0%, *p* = 0.05) and coronary artery disease (4.6% vs. 2.0%, *p* = 0.06) prevalence was noted in the post-P group (Table 1).

The univariate analysis, comparing pre-P to post-P, demonstrated a significant reduction in overall VTE rates in the post-P group (1.3%) compared to the pre-P group (4.0%), [OR = 0.31 (95% CI: 0.10–0.97; *p* = 0.03)], indicating a 69% reduction in the odds of VTE. However, specific components like DVT (1.4% vs. 0.3%; *p* = 0.14) and PE (1.4% vs. 0.3%; *p* = 0.14) showed non-significant trends toward improvement. Patients who had concomitant DVT and PE were counted separately and were identical between the groups (0.3% vs. 0.3%; *p* = 0.92). The time to diagnose VTE was post-admission day 4 and day 8 (2 PE), day 11 (1 DVT), and day 4 (1 concomitant DVT and PE).

The rates of bleeding episodes that occurred after the initiation of the VTEp were slightly higher in the post-P group (1.6%) compared to the pre-P group (0.9%), but this difference was not statistically significant (OR 1.9, 95% CI: 0.45–8.13; *p* = 0.36) (Table 2).

**Table 2:** Univariate analysis of VTE rates and bleeding in Pre-P and Post-P groups

All Patients	Pre-*P* (*n* = 350)	Post-*P* (*n* = 305)	OR (95% CI)	*P*-value
Overall VTE rate	14 (4.0%)	4 (1.3%)	0.31 (0.10–0.97)	0.03
Deep Vein thrombosis (DVT)	5 (1.4%)	1 (0.3%)	0.22 (0.02–1.95))	0.14
Pulmonary Embolism (PE)	8 (2.3%)	2 (0.7%)	0.28 (0.05–1.3)	0.09
Concomitant DVT and PE	1 (0.3%)	1 (0.3%)	1.14 (0.07–18.43)	0.92
Bleeding	3 (0.9%)	5 (1.6%)	1.9 (0.45–8.13)	0.36

**Fig. 3:**
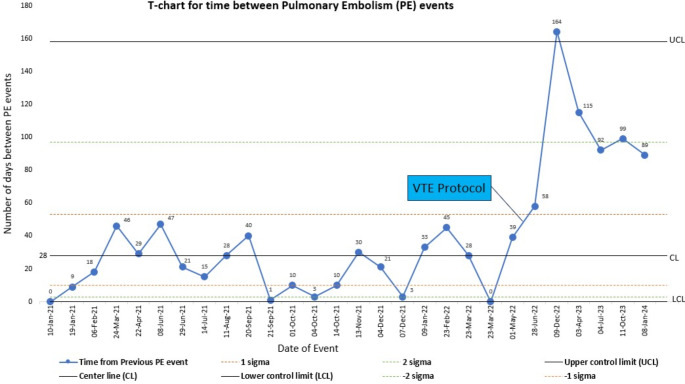
Shewhart Control T chart

The Shewhart control T chart showed significant prolongation of the time intervals between two consecutive PE events (Fig. 3).

In the group of patients who were not compliant with the new protocol after its implementation (*n* = 125), the rates were as follows: VTE: 4 (3.2%), PE = 3 (2.4%), and DVT: 2 (1.6%).

Sub-analysis of patients with severe TBI (AIS 3–5) in the pre-implementation (*n* = 161) and post-implementation (*n* = 127) groups was done. Demographic parameters such as age, gender distribution, and BMI were similar between the groups. Baseline laboratory parameters showed no significant differences, including hemoglobin, platelets, INR, PTT, and fibrinogen levels. ISS were comparable (23.6 ± 9.7 vs. 23.3 ± 11.0). The Head AIS score was significantly lower in the post-implementation group (3.6 ± 0.9 vs. 3.8 ± 0.9, *p* = 0.02). Patients in the post-implementation group were more likely to have spinal cord injuries (23.6% vs. 13.7%; *p* = 0.02), while other injury patterns, including unstable spinal fractures, pelvic fractures, and long bone fractures, showed no significant differences. Ventilatory days, TICU length of stay, and total hospital LOS were comparable. The time to initiate chemoprophylaxis was shorter in the post-implementation group (50.6 vs. 57.9 h) and was not statistically significant (Table 3).

**Table 3:** Sub-analysis for pre-implementation and post-implementation groups in patients with severe TBI (*n* = 288)

Parameters	Pre-implementation TBI (*n* = 161)	Post-implementation TBI (*n* = 127)	*P*-Value
Age	36.6 ± 14.9	35.7 ± 14.4	0.57
Gender			
Male	151 (93.8%)	121 (95.3%)	0.58
Female	10 (6.2%)	6 (4.7%)	
Body Mass Index (BMI)	26.3 ± 4.7	26.0 ± 4.3	0.64
**Laboratory parameters at Baseline**			
Hemoglobin	13.3 ± 2.1	12.9 ± 1.9	0.11
Platelets	250 ± 73	236 ± 68	0.09
INR	1.10 ± 0.1	1.14 ± 0.2	0.06
PTT	26.2 ± 5.4	25.7 ± 4.9	0.42
Fibrinogen	2.6 ± 1.1	2.5 ± 1.0	0.52
Lactate at admission	2.5 ± 1.9	2.5 ± 1.5	0.82
Base Excess (BE) at admission	-2.5 ± 4.2	-2.8 ± 3.6	0.40
ROTEM baseline abnormal	40 (25.8%)	28 (23.7%)	0.69
**Blood transfusion within 24 h**			
PRBC	16 (9.9%)	7 (5.5%)	0.16
FFP	6 (3.7%)	5 (3.9%)	0.92
Platelet	3 (1.9%)	5 (3.9%)	0.28
TXA	38 (23.6%)	25 (20.2%)	0.48
PCC	1 (0.6%)	2 (1.6%)	0.41
Fibrinogen	23 (14.3%)	21 (16.9%)	0.53
Prehospital time	86 ± 52	97 ± 84	0.19
SBP at admission			0.47 for all
< 90	17 (10.6%)	10 (8.1%)	
≥ 90	144 (89.4%)	114 (91.9%)	
ISS	23.6 ± 9.7	23.3 ± 11.0	0.79
Head AIS	3.8 ± 0.9	3.6 ± 0.9	0.02
Spinal cord injury	22 (13.7%)	30 (23.6%)	0.02
Unstable Spinal Fracture	8 (5.0%)	6 (4.7%)	0.92
Pelvic fracture	21 (13.0%)	22 (17.3%)	0.31
Long bone fracture	26 (16.1%)	21 (16.5%)	0.93
Positive pressure ventilation (*n* = 155)			
≤ 5 days	45 (51.1%)	40 (59.7%)	0.28 for all
> 5 days	43 (48.9%)	27 (40.3%)	
Ventilator days	3 (1–87)	3 (1–76)	0.71
LOS in TICU	4 (1–65)	3 (1–34)	0.22
Total hospital LOS	12 (1–60)	13 (1-100)	0.80
Time to initiate chemoprophylaxis (hours)	57.9 (48.8–67.1)	50.6 (46.0-55.1)	0.12
VTEp interruptions	1.5 (1–33)	4 (1–12)	0.45

**Abbreviations**: INR: The international normalized ratio; PTT: partial thromboplastin time; TBI: Traumatic brain injury; AIS: Abbreviated Injury Scale; DVT: Deep vein thrombosis; LOS: Length of stay; ICU: Intensive Care Unit; ROTEM: Rotational thromboelastometry; VTEp: Venous thromboembolism post-implementation protocol; pRBC: packed red blood cells

VTE rates and intracranial bleeding in patients with TBI were compared between the pre-implementation and post-implementation groups (Table 4). The overall VTE rate was lower in the post-implementation group (1.6% vs. 4.3%, OR: 0.35, 95% CI: 0.07–1.72), but the difference was not statistically significant (*p* = 0.17). No cases of DVT were observed in the post-implementation group, compared to 1.9% in the pre-implementation group (*p* = 0.12). PE rates and concomitant DVT and PE rates were similar between groups. Intracranial bleeding occurred more frequently in the post-implementation group (1.6% vs. 0.6%), but this difference was not statistically significant (*p* = 0.42). (Table 4).

**Table 4:** Univariate analysis of VTE rates and intracranial bleeding in patients with Pre-P and Post-P groups for patients with TBI

All Patients	Pre-implementation (*n* = 161)	Post-implementation (*n* = 127)	OR (95% CI)	*P*-value
**Overall VTE rate**	7 (4.3%)	2 (1.6%)	0.35 (0.07–1.72)	0.17
DVT	3 (1.9%)	0 (0.0%)	-	0.12
PE	3 (1.9%)	1 (0.8%)	0.41 (0.04–4.06)	0.43
Both	1 (0.6%)	1 (0.8%)	1.27 (0.07–20.50)	0.86
Intracranial bleeding	1 (0.6%)	2 (1.6%)	2.56 (0.23–28.5)	0.42

Out of 305 patients in the post-P group, 253 (83%) had a timely sampling for the anti-Xa test. 102 (40.4%) had an initial level below 0.2 IU/ml, 128(50.6%) had levels between 0.2 and 0.4, and 23(9%) were above 0.4. The analysis of the anti-Xa levels (< 0.2 vs. ≥0.2) in the post-P cohort revealed notable differences in certain parameters. Patients with Anti-Xa levels ≥ 0.2 had a significantly lower mean weight (73.7 ± 13.4 kg vs. 78.1 ± 15.2 kg, *p* = 0.01) and BMI (25.9 ± 3.8 vs. 27.1 ± 4.2, *p* = 0.02) compared to those with Anti-Xa levels < 0.2. Additionally, the enoxaparin dose per kilogram was slightly higher in the Anti-Xa ≥ 0.2 group (0.43 ± 0.07 vs. 0.41 ± 0.07, *p* = 0.02). Other parameters, such as age, gender distribution, ISS, creatinine clearance, and total enoxaparin dose, were similar between groups. (Table 5).

**Table 5:** Analysis of the anti-xa levels (< 0.2 vs. ≥0.2) in the post-P cohort (*n* = 253)

	Anti Xa < 0.2 (*n* = 102)	Anti Xa ≥ 0.2 (*n* = 151)	*P* value
**Age years (mean ± SD)**	37.4 ± 13.4	36.3 ± 13.7	0.53
**Males**	94 (92.2%)	143 (94.7%)	0.41
**ISS (mean ± SD)**	20.6 ± 10.2	18.4 ± 9.1	0.06
**Weight (mean ± SD)**	78.1 ± 15.2	73.7 ± 13.4	0.01
**BMI (mean ± SD)**	27.1 ± 4.2	25.9 ± 3.8	0.02
**Creatinine clearance**	119.4 ± 16.3	118.5 ± 20.6	0.70
**Enoxaparin Dose (mean ± SD)**	30.8 ± 3.2	30.6 ± 2.4	0.56
**Enoxaparin/kg (mean ± SD)**	0.41 ± 0.07	0.43 ± 0.07	0.02

### Post hoc power analysis

The difference between two independent proportions and the effect size was calculated using baseline (4%) and expected rates (1.3%). The anticipated effect size was 0.146, representing the predicted reduction in VTE incidence from 4 to 1.3% with the new intervention, and a significance level (α) set at 0.05, with a sample size of 350. Running this calculation, the study exhibited a power of approximately 0.909, suggesting a 90.9% chance of detecting a significant difference in VTE incidence.

## Discussion

In a cohort of patients admitted to the TICU, implementation of a VTEp protocol with 30 mg enoxaparin twice daily and dose calibration to peak anti-Xa levels reduced the incidence of VTE without increasing the risk of bleeding in comparison to a fixed-dose dalteparin regimen. Although standard statistical analysis did not reach significance for PE or DVT when analyzed separately, the Shewhart control T chart proved significant prolongation in the time interval between two consecutive PE events after the new protocol was implemented, reflecting a decrease in the occurrence of PE. In the subgroup of severe TBI patients studied, VTE trended down without an increase in the incidence of post-prophylaxis intra-cranial bleeds.

Although dalteparin and enoxaparin are not pharmacodynamically identical, large-scale clinical studies have suggested that both agents were clinically equipotent in VTEp [11–13]. Switching from once daily dalteparin dose to twice daily enoxaparin was because obtaining a steady state Anti-Xa level after the third consecutive dose of a drug would mean that a much longer time would pass before a level is obtained if dalteparin is used. Currently, anti-Xa trauma literature is based on enoxaparin prophylaxis. Theoretically, since trauma patients are at elevated risk for both VTE and bleeding, optimizing the dose of LMWH as per the measured level of factor-Xa inhibition would increase its protective efficacy against VTE while avoiding over anti-coagulation, thus promoting its safety.

In the cohort in this study, the initial enoxaparin dose was 30 mg with dose calibration as per anti-Xa level. In the past decade, using this test for routine monitoring and dose titration in trauma settings has shown variable results. Singer et al. suggested that Anti-Xa guided enoxaparin dosing decreased the rate of DVT in high-risk trauma patients [14]. Ko et al. had suggested that dose calibration may decrease VTE incidence [15]. Karcutskie et al. found that the rates of VTE were not reduced with anti-Xa-guided dosing before and after propensity-matched analysis (*n* = 132 control fixed dosing and *n* = 84 anti-Xa adjustment group) [16]. Moreover, despite repeated increases in the enoxaparin dosing, almost 50% of patients did not reach a prophylactic anti-Xa level. However, other studies performed in similar pre-and post-comparisons using Anti Xa guidance along with a 40 mg initial dose had shown improved outcomes [17, 18]. In a systematic review with meta-analysis, Verhoeven et al. [19] concluded that the incidence of VTE was lower with a higher initial anti-Xa level, but later adjustment of the dose after an initial low level was not. However, the study had important limitations as the patients with initial low anti-Xa levels also had prolonged time before VTEp was initiated. Furthermore, the studies in this meta-analysis were of moderate quality. Although a 40 mg initial dosing of enoxaparin is now recommended by trauma associations for non-TBI patients younger than 65 years old and with creatinine clearance above 60 ml/m, to the author’s knowledge, there are no prospective randomized studies comparing 30 and 40 mg dosages. Furthermore, concurrent large-scale prospective studies are still using 30 mg as the initial enoxaparin dose, namely, the CLOTT registry and Prevent-Clot [20–22].

In this cohort, the analysis of the anti-Xa levels suggests that an initial 30 mg dose may have been sub-optimal. Of the 253 patients who had timely anti-Xa tests, 102 (40.4%) had an initial level below 0.2 IU/ml, of whom 3 (3%) developed VTE. Meanwhile, among 151 patients (59.6%) with levels 0.2 IU/ml and above, only 1 (0.7) had VTE. Weight and BMI were higher in patients who had an initial low anti-Xa level, emphasizing the strategy of weight-based adjustment. This weight-based strategy was advocated to obtain higher anti-Xa levels and was later challenged by showing no benefit in decreasing the VTE rate [23–25]. On the other hand, concerning the bleeding episodes that occurred after VTEp, five patients bled in the post-P group, of whom two patients with severe TBI had progression of intracranial bleeding. Anti-Xa levels were in the desired range in three patients, high in one, and not done in one. The two intracranial bleeds were catastrophic, leading to craniectomy and permanent deterioration in brain function. Those bleeds could not be ascertained to be secondary to VTEp or the natural progression of the original trauma. The three other bleeding episodes were hemothorax with rib fractures that required thoracoscopy, hematuria in a patient with renal injury requiring blood transfusion, and a patient who dropped his hemoglobin secondary to pelvic hematoma expansion and was treated conservatively. A correlation between the occurrence of post-prophylaxis bleeding and supra-therapeutic anti-Xa level could not be drawn.

Trauma research currently emphasizes the initiation of VTEp before 48 h of admission [26–29]. In the post-implementation phase of this study, the time to initiate prophylaxis was decreased from 49.3 h (43.9–54.6) in the Pre-P group to 43.7 h (40.9–46.4). This change was not statistically significant, but the downward trend may have contributed to the improvement. In examining the four VTEs in the present study, the time of initiation was relatively delayed, 45–49 h post admission, and the anti-Xa level was low or borderline (0.1 (PE), 0.18 (PE), 0.19 (DVT) & 0.21(concomitant DVT and PE case)). In 2 cases the results of anti-Xa were obtained after the VTE was diagnosed. None of those 4 patients had dose adjustment. Obtaining timely and correct peak anti-Xa levels has been described as low [25, 30]. In this study, correct sampling was obtained in 83% of the post-P group. Nevertheless, we acknowledge that strict compliance with the above protocol was challenging, especially titration and documenting the reasons for the delay in initiating VTEp. Better execution of the protocol may be achieved by automation and having a protocol that is partly nurse driven.

In real life, compliance with any protocol could be better. In this project, 29% of patients did not follow the new protocol after its implementation and continued the previous dalteparin-based regimen. Those patients developed VTE in the same frequency as the pre-P group. Nevertheless, according to the Trauma Performance Improvement Program (TQIP) biannual benchmark report, our Trauma Center ranking in PE events improved from the 10th decile in the spring and fall of 2022 to the 9th percentile in spring 2023, then the 3rd percentile in fall 2023 and spring 2024 [31].

This study is the first to compare fixed-dose dalteparin-based VTEp in trauma with anti-Xa-guided enoxaparin prophylaxis. Being a single-center retrospective study, the results cannot be generalized, but they add to the growing knowledge in this field, both in general trauma and severe TBI patients.

### Limitations

The exclusion of 29% of the post-protocol population after it had started significantly decreased the study population. The decision to exclude them was made by individual trauma or neurosurgery attendings who decided to wait until the results of this study are obtained before accepting the new protocol. More education has been initiated, and the rate of this non-compliance is continuing to decrease. As for anti-Xa testing, although compliance was relatively high with the initial testing, follow-up testing after dose adjustments was low. Another limitation is that VTEp was standardized to hospitalized patients only, and post-discharge clinical follow-up was limited to one month. Furthermore, the study results were gender biased, as 94% of the patients were men, but this reflected the real demographics of trauma in the country [32, 33]. Reaching a statistically significant reduction only in the incidence of VTE and not in PE or VTE separately, and not reaching any significance in the TBI subgroup analysis, are likely to be secondary to the limited number in the population studied. Larger and multicenter studies are needed for such an uncommon disease.

### Conclusion

This study suggests that in a cohort of injured patients in the ICU, one-time enoxaparin dose adjustment guided by initial anti-Xa level is associated with less VTE than fixed-dose dalteparin prophylaxis. There is no increase in the incidence of bleeding episodes. However, prospective studies are required to support the study findings.

## Data Availability

All data are presented in the manuscript, figures, and tables. It will be available upon reasonable request and after approval of the medical research center of Hamad Medical Corporation after signing a data-sharing agreement form.
